# Duck plague virus UL41 protein inhibits RIG-I/MDA5-mediated duck IFN-β production via mRNA degradation activity

**DOI:** 10.1186/s13567-022-01043-y

**Published:** 2022-03-18

**Authors:** Tianqiong He, Mingshu Wang, Anchun Cheng, Qiao Yang, Ying Wu, Renyong Jia, Shun Chen, Dekang Zhu, Mafeng Liu, Xinxin Zhao, Shaqiu Zhang, Juan Huang, Bin Tian, Xumin Ou, Sai Mao, Di Sun, Qun Gao, Yanling Yu, Ling Zhang, Yunya Liu

**Affiliations:** 1 Institute of Preventive Veterinary Medicine, Sichuan Agricultural University, Wenjiang, 611130 China; 2 Avian Disease Research Center, College of Veterinary Medicine, Sichuan Agricultural University, Wenjiang, 611130 China; 3 Key Laboratory of Animal Disease and Human Health of Sichuan Province, Sichuan Agricultural University, Wenjiang, 611130 China

**Keywords:** DPV, UL41 protein, IFN-β, RLRs, mRNA, innate immune response

## Abstract

Retinoic acid-inducible gene I (RIG-I)-like receptors (RLRs) are cytosolic pattern recognition receptors that initiate innate antiviral immunity. Recent reports found that duck RLRs significantly restrict duck plague virus (DPV) infection. However, the molecular mechanism by which DPV evades immune responses is unknown. In this study, we first found that the DPV UL41 protein inhibited duck interferon-β (IFN-β) production mediated by RIG-I and melanoma differentiation-associated gene 5 (MDA5) by broadly downregulating the mRNA levels of important adaptor molecules, such as RIG-I, MDA5, mitochondrial antiviral signalling protein (MAVS), stimulator of interferon gene (STING), TANK-binding kinase 1 (TBK1), and interferon regulatory factor (IRF) 7. The conserved sites of the UL41 protein, E229, D231, and D232, were responsible for this activity. Furthermore, the DPV CHv-BAC-ΔUL41 mutant virus induced more duck IFN-β and IFN-stimulated genes (Mx, OASL) production in duck embryo fibroblasts (DEFs) than DPV CHv-BAC parent virus. Our findings provide insights into the molecular mechanism underlying DPV immune evasion.

## Introduction

Retinoic acid-inducible gene I (RIG-I)-like receptors (RLRs), including RIG-I, MDA5 and laboratory of genetics and physiology 2 (LGP2), are critical cytosolic RNA sensors that trigger the innate immune response [1]. Both RIG-I and MDA5 consist of two N-terminal cysteine/aspartic protease (caspase) and activation and recruitment domains (CARDs), a helicase domain, and a C-terminal domain (CTD). Upon sensing diverse cytosolic double-stranded RNAs (dsRNAs), RIG-I undergoes conformational changes, oligomerization, and exposure of the N-terminal CARD domains to interact with the CARD domain of a signalling adaptor, mitochondrial antiviral signalling protein (MAVS) [2]. MAVS transmits signals to downstream signalling molecules, the IκB kinase (IKK)-related kinases TBK1 and IKKε, and TBK1 and IKKε then phosphorylate IRF3/7 and NF-κB and induce their nuclear translocation, resulting in inflammatory cytokine and interferon (IFN) production [3]. In addition, STING is a central adaptor molecule that links DNA- and RNA-sensing pathways to activate IFN-β production. STING also interacts with RIG-I and MAVS, but not with MDA5, to form a stabilized complex upon virus infection [4]. Therefore, STING plays a critical role in RIG-I-mediated antiviral signalling [5, 6]. IFNs are classified into three types (type I, type II, and type III), among which type I IFNs (including a multigene α subtype with 13 members in humans and single β, ε, κ, τ, δ, and ω genes) are quintessential antiviral cytokines because of their central roles in the antiviral immune response, and IFN-α/β in birds and mammals are not grouped together in the phylogenetic tree [7].

Duck plague is an acute and contagious fatal disease with high morbidity and mortality in domestic waterfowl which causes substantial economic losses in the commercial waterfowl industry [8–16]. DPV, the causative agent of infectious disease, belongs to the *Alphaherpesvirinae* subfamily [17]. The DPV virion is composed of an envelope, a tegument layer and a spherical nucleocapsid that contains double-stranded DNA [18, 19]. Alphaherpesviruses encode 23 tegument proteins that play roles in promoting viral replication and viral assembly, regulating viral and host protein synthesis, and immune evasion [20].

Host shutoff has emerged as a key process that facilitates the reallocation of cellular resources for viral replication and evasion of host antiviral immune responses [21]. The virion host shutoff (VHS) protein, encoded by the herpes simplex virus-1 (HSV-1) *UL41* gene, is a late tegument protein and an endoribonuclease with a substrate specificity for ribonuclease (RNase) A [22]. The VHS protein specifically degrades a wide array of mRNAs and induces the rapid shutoff of host protein synthesis [23, 24]. On the one hand, the VHS protein facilitates the turnover of all kinetic classes of viral mRNAs [25]. On the other hand, the VHS protein plays crucial roles in escaping the host innate immune response. The VHS protein downregulates the expression of major histocompatibility complex (MHC) class I/II molecules, impairs antigen presentation [26–28], suppresses the production of proinflammatory chemokines and cytokines, inactivates human monocyte-derived dendritic cells [21, 29], and degrades the mRNAs of several IFN-stimulated genes (ISGs), such as ch25h [30], ZAP [31], tetherin [32], viperin [33], IFIT3 [34] and TNF-α [35].

The duck RIG-I, MDA5 and STING proteins play pivotal roles in the innate immune response of the host to DPV infection [36–38]. In addition, DPV is immunosuppressive and exhibits broad cell tropism [39, 40]. While knowledge on the molecular mechanism of DPV immune evasion is limited, the UL47 protein is known to interact with signal transducer and activator of transcription 1 (STAT1) to inhibit duck IFN-β production [41]. In this study, we found that the DPV UL41 protein abrogated RIG-I/MDA5-mediated duck IFN-β production by downregulating the mRNA levels of important adaptors, and the conserved sites of the UL41 protein, E229, D231, and D232, were responsible for this activity. Our findings provide new insights into host-virus interactions and contribute to the development of new antiviral drugs.

## Materials and methods

### Viruses, cells, and vectors

The DPV CHv-BAC [42] and DPV CHv-BAC-ΔUL41 [43] recombinant viruses were blindly passaged up to 10 passages in DEFs. Passage 10 viruses were used for all experiments described in the manuscript.

DEF cells were cultured in minimal essential medium (MEM; Gibco, Meridian Road Rockford, USA) supplemented with 10% (v/v) foetal bovine serum (FBS; Gibco, Meridian Road Rockford, USA) at 37 °C and 5% CO_2_. For viral infections, maintenance medium supplemented with 2% FBS was added. Commonly used reagents were prepared in our laboratory. HEK293T cells were cultured in RPMI-1640 medium supplemented with 10% FBS at 37 °C and 5% CO_2_ [43].

All primers were designed by Primer Premier 5 software (Table 1). The recombinant plasmids pcaggs-MDA5-Flag, pcaggs-MAVS-Flag, pcaggs-STING-Flag, pcaggs-TBK1-Flag, pcaggs-IRF7-Flag, pcaggs-UL41-HA, pcaggs-mUL41-HA and IFN-β-Luc express firefly luciferase under the control of the duck IFN-β promoter (−96 to +1) were prepared in our laboratory [38, 43, 44]. The entire RIG-I open reading frame (ORF) (accession no. KC869660.1) was inserted into the pcaggs vector to generate pcaggs-RIG-I-Flag. The pRL-TK internal control vector and pGL4.45 vector were purchased from Promega.

**Table 1:** **Sequences and primer pair characteristics.**

Primer	Plasmid	Primer sequence (5′ → 3′)	Accession No.
P_1_	pcaggs-IRF7-Myc	CATCATTTTGGCAAAGAATTCGCCACCATGGCAGCGGCGGAGAGCGAAG	MG707077.1
P_2_		GGCAGAGGGAAAAAGATCTTCACAGGTCCTCCTCTGAGATCAGCTTCTGCTCGTCTATCTGCATGTTGTACTGCTCG	
P_3_	pcaggs-RIG-I-Flag	CATCATTTTGGCAAAGAATTCGCCACCATGACGGCGGACGAGAAGCGG	MK636873.1
P_4_		AAAAAGATCTGCTAGCTCGAGCTACTTATCGTCGTCATCCTTGTAATCGATCTTATCGTCGTCATCCTTGTAATCTCCCTTATCGTCGTCATCCTTGTAATCAAATGGTGGGTACAAGTT	
P_5_	duck RIG-I (RT-PCR)	TCTCTGTCGGTCGGATAA	KC869660.1
P6		TCATCAGGTTCTGCTTCTTC	
P7	duck MDA5 (RT-PCR)	GCTGAAGAAGGCCTGGACAT	KJ451070.1
P8		TCCTCTGGACACGCTGAATG	
P9	duck MAVS (RT-PCR)	AGCCCAGAAATGAACCCCAG	KX290106.1
P10		TCGAACTGCTGCTGGATGAG	
P11	duck STING (RT-PCR)	CCACATCTTGATCCCGCTGA	XM_013100766.1
P12		ATTGCGTAGAGGCTGTGCTT	
P13	duck TBK1 (RT-PCR)	TGATCTATGAAGGTCGGCGTTT	MG772817.1
P14		CTGCTCACTACGAAGATAGGATTCTC	
P15	duck IRF7 (RT-PCR)	CGCCACCCGCCTGAAGAAGT	MG707077.1
P16		CTGCCCGAAGCAGAGGAAGAT	
P17	duck IFN-β (RT-PCR)	TCTACAGAGCCTTGCCTGCAT	KM035791.2
P18		TGTCGGTGTCCAAAAGGATGT	
P19	duck OASL (RT-PCR)	TCTTCCTCAGCTGCTTCTCC	KY775584.1
P20		ACTTCGATGGACTCGCTGTT	
P21	duck Mx (RT-PCR)	TGCTGTCCTTCATGACTTCG	NM_001310409.1
P22		GCTTTGCTGAGCCGATTAAC	
P23	UL41(RT-PCR)	TGATTTACACCGCTACCCTA	AFC61841.1
P24		TCTCACTTCTTTCAGCCATT	
P25	pGPU6/GFP/Neo-shIRF7	GCTCATCGAGCAGTACAACAT	
P26	pGPU6/GFP/Neo-shNC	TTCTCCGAACGTGTCACGT	

### RT-qPCR

Total RNA was extracted from DEF cells using TRIzol reagent l (Invitrogen, CA, USA) according to the manufacturer’s recommendations, and complementary DNA (cDNA) was generated using the PrimeScript® RT reagent kit with gDNA Eraser (Takara, Dalian, China). Target genes were detected using previously described primers (Table 1), and the threshold cycle (Ct) values were normalized to that of 18S rRNA. RT-qPCR was performed according to the manufacturer’s protocol, and the reaction mixture was comprised of the following components: 10 μL of SYBR Green I Mix, 2 μL of each primer, 2 μL of standard template, and autoclaved double-filtered nanopure water added to a final volume of 20 μL. Amplification reactions were performed with preliminary denaturation at 95 °C for 1 min, followed by 40 cycles of denaturation at 95 °C for 20 s, annealing at 60 °C for 30 s and extension at 72 °C for 30 s. All reactions were performed in triplicate with at least three independent experiments. The relative gene expression levels were determined with the 2^−ΔΔ^*C*t method [9].

### Western blotting analysis

Cells were harvested at 36 h post-transfection (hpt). The samples were separated by sodium dodecyl sulphate polyacrylamide gel electrophoresis (SDS-PAGE) and then transferred onto polyvinylidene fluoride (PVDF) membranes. The membranes were blocked with 5% skim milk for 2 h at 37 °C, incubated with a mouse anti-Flag antibody (MBL, Japan, 1:5000), mouse anti-HA antibody (MBL, Japan, 1:4000) or mouse anti-GAPDH antibody (Proteintech, Beijing, 1:20 000) overnight at 4 °C, and then probed with an HRP-conjugated secondary antibody (Bio-Rad, CA, USA) for 1 h at 37 °C. Proteins were detected with Western Blot Chemiluminescence HRP Substrate (Takara, Dalian, China) according to the manufacturer’s instructions [45].

### Indirect immunofluorescence assay (IFA)

HEK293T cells were transfected with pcaggs-UL41-HA, pcaggs-mUL41-HA or empty vector, collected at 36 hpt, fixed with 4% paraformaldehyde overnight at 4 °C, and permeabilized with 0.25% Triton X-100 for 30 min at 4 °C. The cells were rinsed three times with PBST (containing 0.1% Tween-20), blocked with 5% BSA PBS for 2 h at 37 °C, and then incubated with a mouse anti-HA antibody (MBL, Japan, 1:100), goat anti-mouse IgG (H+L) Highly Cross-Adsorbed Secondary Antibody, and Alexa Fluor 488 (Thermo Fisher Scientific, Meridian Road Rockford, USA, 1:1000). All antibodies were diluted in 1% BSA PBS. Finally, cell nuclei were visualized with DAPI (Roche, Mannheim, Germany). Coverslips were sealed with glycerin buffer, and the cells were visualized using a fluorescence microscope (Nikon ECLIPSE 80i, Japan) [46].

### Luciferase reporter assay

DEF cells were co-transfected with IFN-β-Luc (400 ng/well) and the internal control pRL-TK (4 ng/well) together with the specific expression plasmid (400 ng/well), pcaggs-UL41-HA (400 ng/well), pcaggs-mUL41-HA or empty vector using Lipofectamine 3000 (Invitrogen, CA, USA) according to the manufacturer’s instructions. The cells were harvested at 36 hpt, and firefly luciferase activity was measured by the dual-luciferase assay system (Promega) according to the manufacturer’s instructions [47].

### Tissue culture infectious dose 50 (TCID_50_)

DEF cells were infected with DPV CHv-BAC or DPV CHv-BAC-ΔUL41 at an MOI of 5 and incubated at 37 °C for 2 h. The culture supernatant of virus-infected cells was discarded, and maintenance medium supplemented with 2% FBS was added. At 4 h post-infection (hpi), the cytoplasmic samples were washed twice with PBS and collected to determine the TCID_50_ using tenfold serial dilutions. All samples were tested in triplicate with at least three independent experiments.

### Statistical analysis

Different groups were compared with one-way ANOVA using GraphPad Prism 7.0 software (La Jolla, CA, USA). All experiments were repeated at least three times independently. The data are expressed as the means and standard error of the mean (SEM). Asterisks indicate the level of statistical significance (*p < 0.05; **p < 0.01; ***p < 0.001; ****p < 0.0001).

## Results

### The DPV UL41 protein inhibited duck IFN-β signalling activation induced by poly(I:C) stimulation

**Figure 1:**
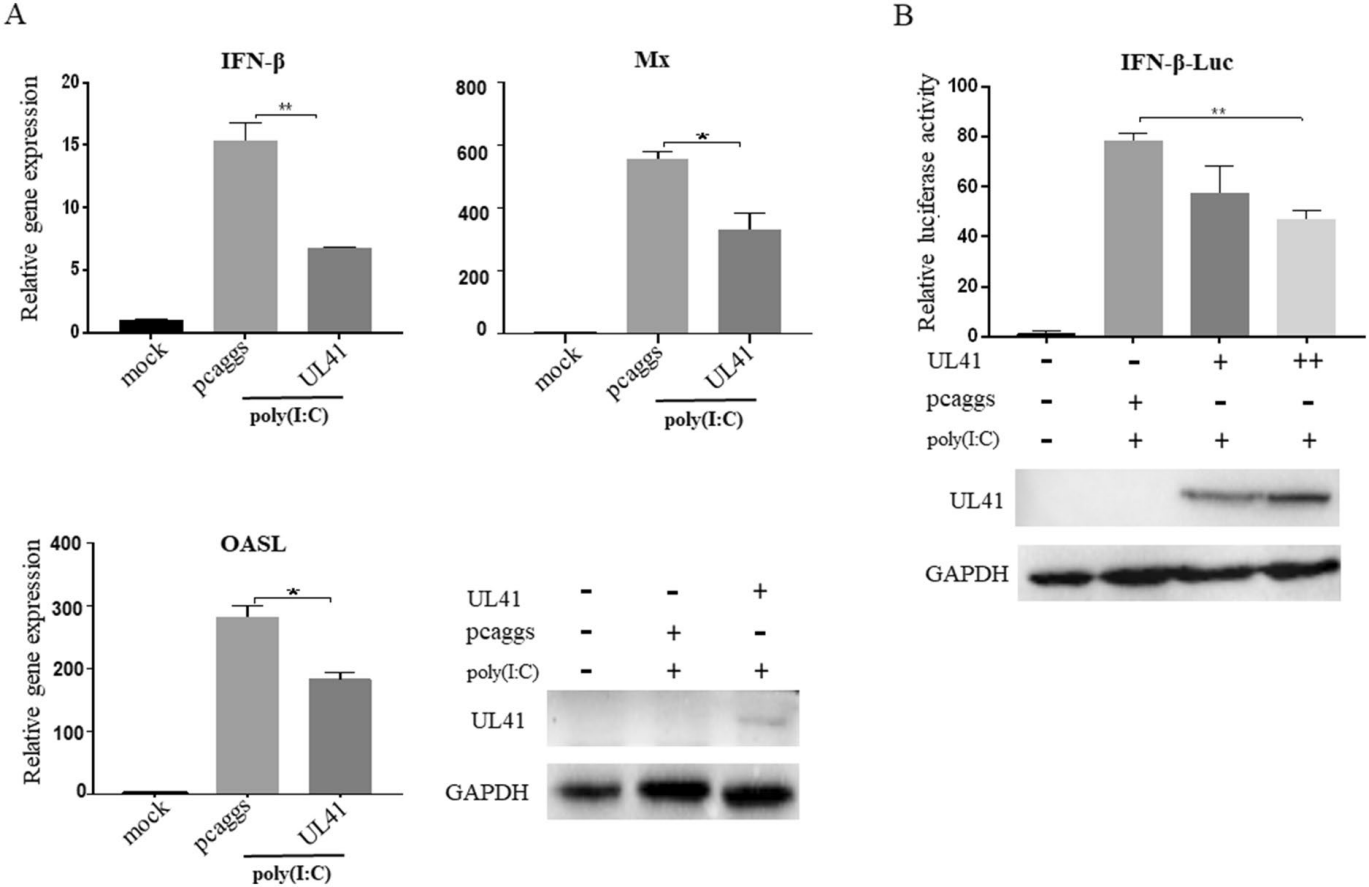
**The DPV UL41 protein inhibited duck IFN-β signalling activation induced by poly(I:C).** All transfected samples were collected at 36 hpt. **A** The DPV UL41 protein inhibited duck IFN-β production induced by poly(I:C) in DEF cells. DEF cells were transfected with pcaggs-UL41-HA or empty vector and then stimulated with 50 µg/mL poly(I:C) after 12 hpt. The cells were harvested and detected by RT-qPCR after 24 h of stimulation. **B** The DPV UL41 protein significantly inhibited duck IFN-β-Luc luciferase activity in a dose-dependent manner. DEF cells were co-transfected with the duck IFN-β-Luc luciferase reporter plasmid, pRL-TK, 1 μg/well pcaggs-UL41-HA, 2 μg/well pcaggs-UL41-HA or empty vector and then stimulated with 50 µg/mL poly(I:C) at 12 hpt. The cells were harvested and detected by the dual-luciferase assay at 24 hpt. Protein expression was confirmed by Western blotting. The data were analysed by one-way ANOVA. *p < 0.05, **p < 0.01.

First, we evaluated whether the UL41 protein affects duck IFN-β production. DEF cells were transfected with pcaggs-UL41-HA or an empty vector as a control and then stimulated with the dsRNA analogue poly(I:C). We found that the UL41 protein negatively regulated duck IFN-β and ISG (Mx, OASL) production in DEF cells (Figure 1A). We further co-transfected IFN-β-Luc, pRL-TK, empty vector, or pcaggs-UL41-HA into DEF cells and then stimulated by poly(I:C). As shown in Figure 1B, the UL41 protein significantly inhibited IFN-β-Luc luciferase activity in a dose-dependent manner. These results indicated that the UL41 protein inhibits duck IFN-β production in DEF cells.

### The substitution of crucial sites on the DPV UL41 protein rescued IFN-β signalling

**Figure 2:**
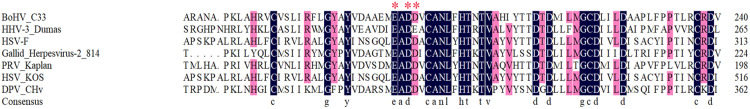
**Comparison of the amino acid sequences of the DPV UL41 protein and its homologues in alphaherpesviruses**.

**Figure 3:**
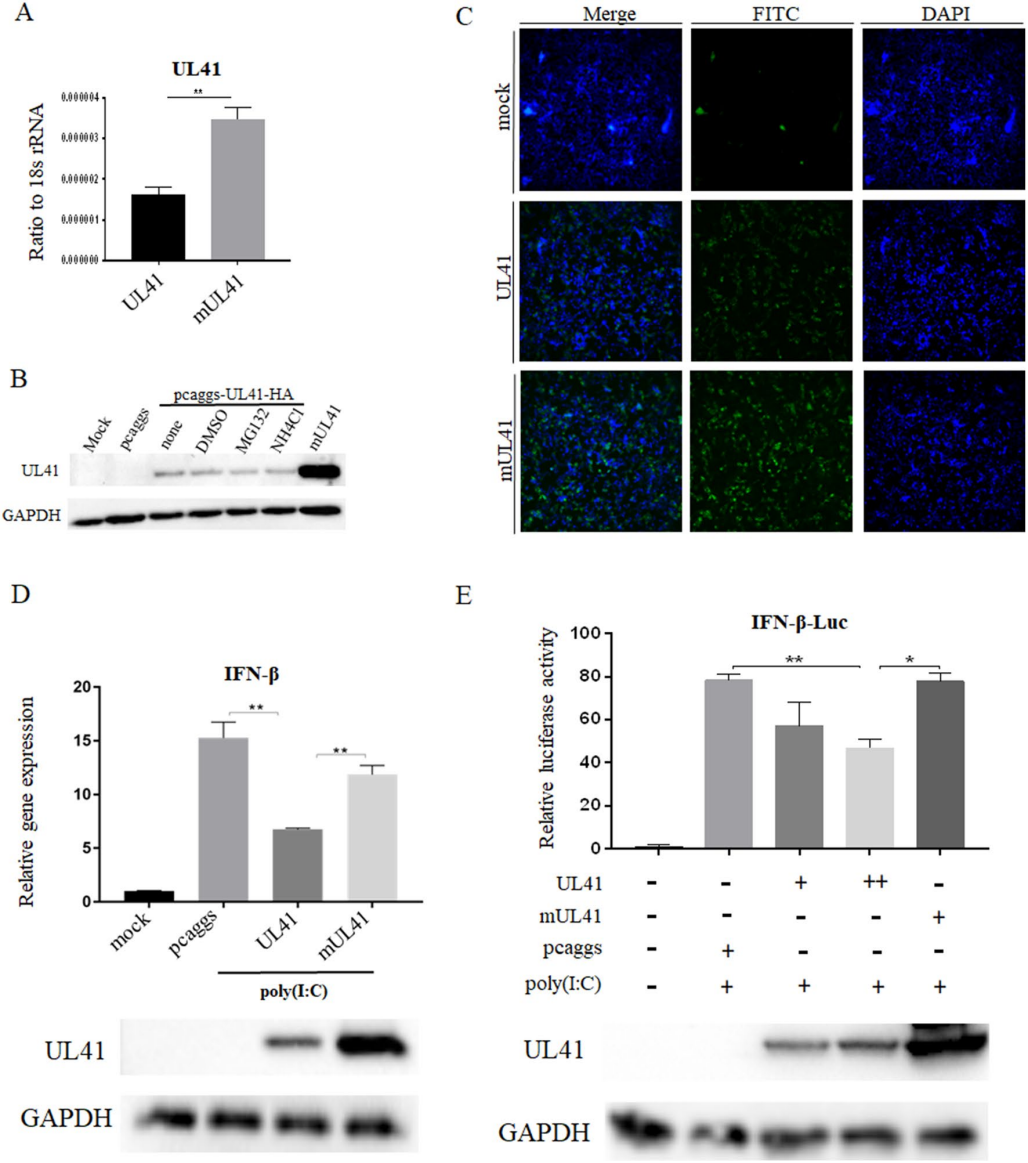
**Mutation of crucial DPV UL41 residues rescued UL41 protein expression and IFN-β signalling.** **A** HEK293T cells were transfected with the pcaggs-UL41-HA, pcaggs-mUL41-HA or empty vector. The cells were then harvested and subjected to RT-qPCR. **B** DEF cells were transfected with pcaggs-UL41-HA, pcaggs-mUL41-HA or empty vector and then treated with DMSO, 10 μM MG132, or 20 mM NH4Cl. **C** HEK293T cells were transfected with the pcaggs-UL41-HA, pcaggs-mUL41-HA or empty vector. Immunofluorescence analysis revealed that the fluorescence (green) was significantly stronger in the mUL41 group than in the UL41 group. **D** DEF cells were transfected with the UL41 or mUL41 expression plasmid or empty vector and then stimulated with 50 µg/mL poly(I:C) at 12 hpt. The cells were harvested and subjected to RT-qPCR after 24 h of stimulation. **E** DEF cells were co-transfected with IFN-β-Luc, pRL-TK, and 1 μg/well pcaggs-UL41-HA, 2 μg/well pcaggs-UL41-HA, 2 μg/well pcaggs-mUL41-HA or empty vector and then stimulated with 50 μg/mL poly(I:C) at 12 hpt. The cells were harvested and detected by the dual-luciferase assay at 24 hpt. Protein expression was confirmed by Western blotting. The data were analysed by one-way ANOVA. *p < 0.05, **p < 0.01.

Based on previous reports [48], genetic and biochemical assays showed that the VHS protein and homologues of other alphaherpesviruses share sequence similarities with a family of nucleases and have some conserved residues that are responsible for RNase activity, such as three amino acid residues of the HSV-1 VHS protein, E192, D194, and D195 [49]. In addition, we compared the DPV UL41 protein with homologues of other alphaherpesviruses and found conserved motifs in the DPV UL41 protein (Figure 2). Based on these findings, we mutated the residues E229, D231, and D232 of the DPV UL41 protein to “alanine (A)”, and we found that the mRNA level of the *UL41* gene was increased in the pcaggs-mUL41-HA group (Figure 3A). In addition, DEF cells were transfected with pcaggs-UL41-HA and then treated with DMSO, the proteasome inhibitor MG132 (10 μM), or the lysosomotrophic neutralizing agent NH4Cl (20 mM). The expression of UL41 protein was not significantly different among these groups, but was increased in the pcaggs-mUL41-HA group, as determined by Western blotting and IFA (Figures 3B and C). These results indicated that the UL41 protein is not subject to lysosomal or proteasomal degradation in transfected cells. We further determined that duck IFN-β production and IFN-β-Luc activity stimulated by poly(I:C) were increased in the mUL41 group (Figures 3D and E).

### The DPV UL41 protein inhibited RIG-I/MDA5-mediated IFN-β-Luc activation

**Figure 4:**
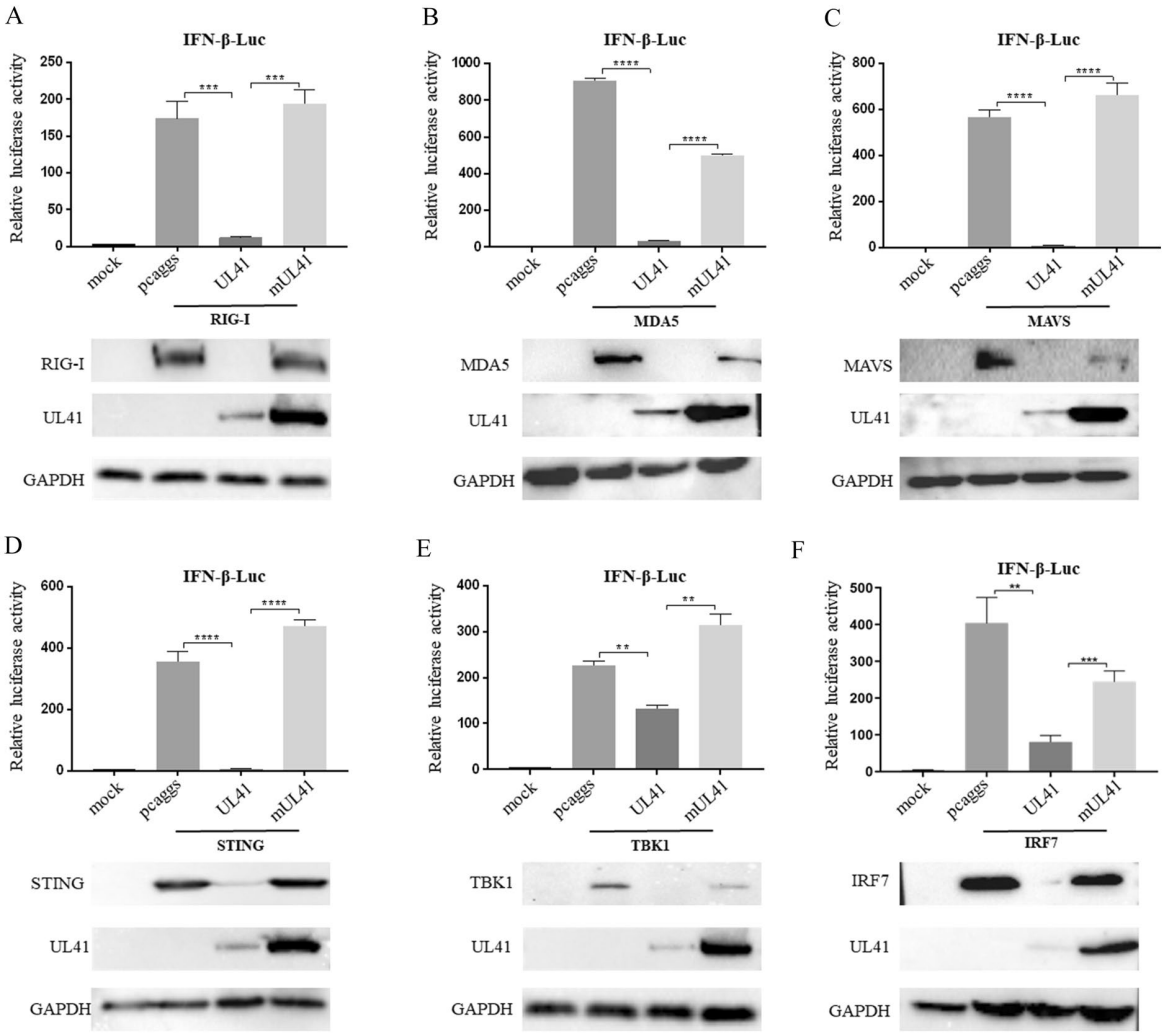
**The DPV UL41 protein inhibited IFN-β-Luc activation by every important adaptor molecule in the RIG-I/MDA5 innate immune pathway.** DEF cells were co-transfected with empty vector, pcaggs-UL41-HA or pcaggs-mUL41-HA together with IFN-β-Luc and plasmids expressing important adaptor proteins in the RIG-I/MDA5 innate immune pathway, namely, RIG-I, MDA5, MAVS, STING, TBK1 and IRF7, and then subjected to luciferase reporter assay to detect IFN-β-Luc promoter activity. Protein expression was confirmed by Western blotting. The data were analysed by one-way ANOVA. *p < 0.05, **p < 0.01, ***p < 0.001 and ****p < 0.0001.

DEF cells were co-transfected with empty vector, pcaggs-UL41-HA or pcaggs-mUL41-HA and IFN-β-Luc along with plasmids expressing important adaptor proteins, including, RIG-I, MDA5, MAVS, STING, TBK1 and IRF7, and IFN-β-Luc luciferase activity was detected [8]. As shown in Figure 4, all the expression constructs resulted in a 150- to 800-fold induction of IFN-β-Luc luciferase activity, and the UL41 protein dramatically reduced duck IFN-β promoter activation mediated by RIG-I, MDA5, MAVS, STING, TBK1 and IRF7. Moreover, the luciferase activity of IFN-β-Luc induced by all the expression constructs was significantly increased in the mUL41 group (Figure 4). Thus, these results showed that the UL41 protein significantly inhibits IFN-β-Luc activation induced by important adaptor molecules.

### The DPV UL41 protein decreased the mRNA levels of important adaptor molecules

**Figure 5:**
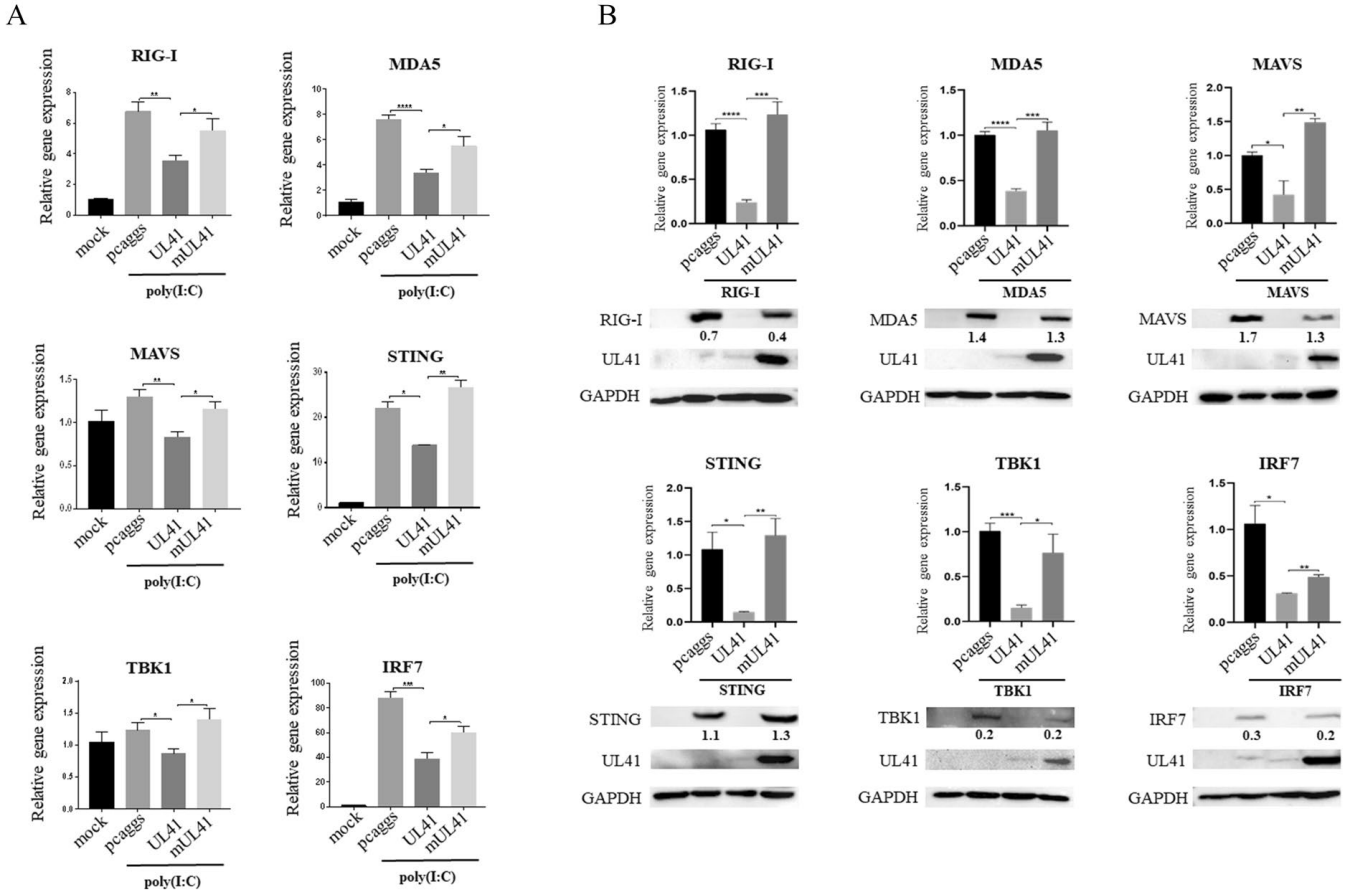
**The DPV UL41 protein broadly decreased the mRNA levels of important adaptor molecules in the RIG-I/MDA5 innate immune pathway.** **A** The DPV UL41 protein inhibited the expression of important adaptor molecules induced by poly(I:C), including, RIG-I, MDA5, MAVS, STING, TBK1 and IRF7, in DEF cells. DEF cells were transfected with pcaggs-UL41-HA, pcaggs-mUL41-HA or empty vector and then stimulated with 50 µg/mL poly(I:C) at 12 hpt. The cells were harvested and detected by RT-qPCR after 24 h of stimulation. **B** DEF cells were co-transfected with pcaggs-UL41-HA, pcaggs-mUL41-HA or empty vector and the RIG-I, MDA5, MAVS, STING, TBK1 and IRF7 expression plasmids. Then, the cells were harvested and subjected to RT-qPCR and Western blot analysis. **C** HEK293T cells were co-transfected with pcaggs-UL41-HA, pcaggs-mUL41-HA or empty vector and the RIG-I, MDA5, MAVS, STING, TBK1 and IRF7 expression plasmids. Then, the cells were harvested and subjected to RT-qPCR and Western blot analysis. The data were analysed by one-way ANOVA. *p < 0.05, **p < 0.01, ***p < 0.001 and ****p < 0.0001.

To further clarify the underlying molecular mechanism, we investigated whether the DPV UL41 protein also affects the mRNA levels of these proteins. As shown in Figure 5A, the transcription of RIG-I, STING, and IRF7 was stimulated by poly(I:C), and the UL41 protein decreased the mRNA levels of various adaptor proteins, especially RIG-I, STING, and IRF7. In addition, DEF cells were co-transfected with the pcaggs-UL41-HA vector or empty vector along with the RIG-I, MDA5, MAVS, STING, TBK1 or IRF7 expression plasmids. We found that the UL41 protein significantly decreased the mRNA and protein levels of RIG-I, MDA5, MAVS, STING, TBK1 and IRF7, which were recovered in the mUL41 group (Figure 5B). These results showed that DPV UL41 protein inhibits duck IFN-β production by broadly degrading mRNAs.

### The DPV UL41 protein inhibits duck IFN-β activation in DEF cells

**Figure 6:**
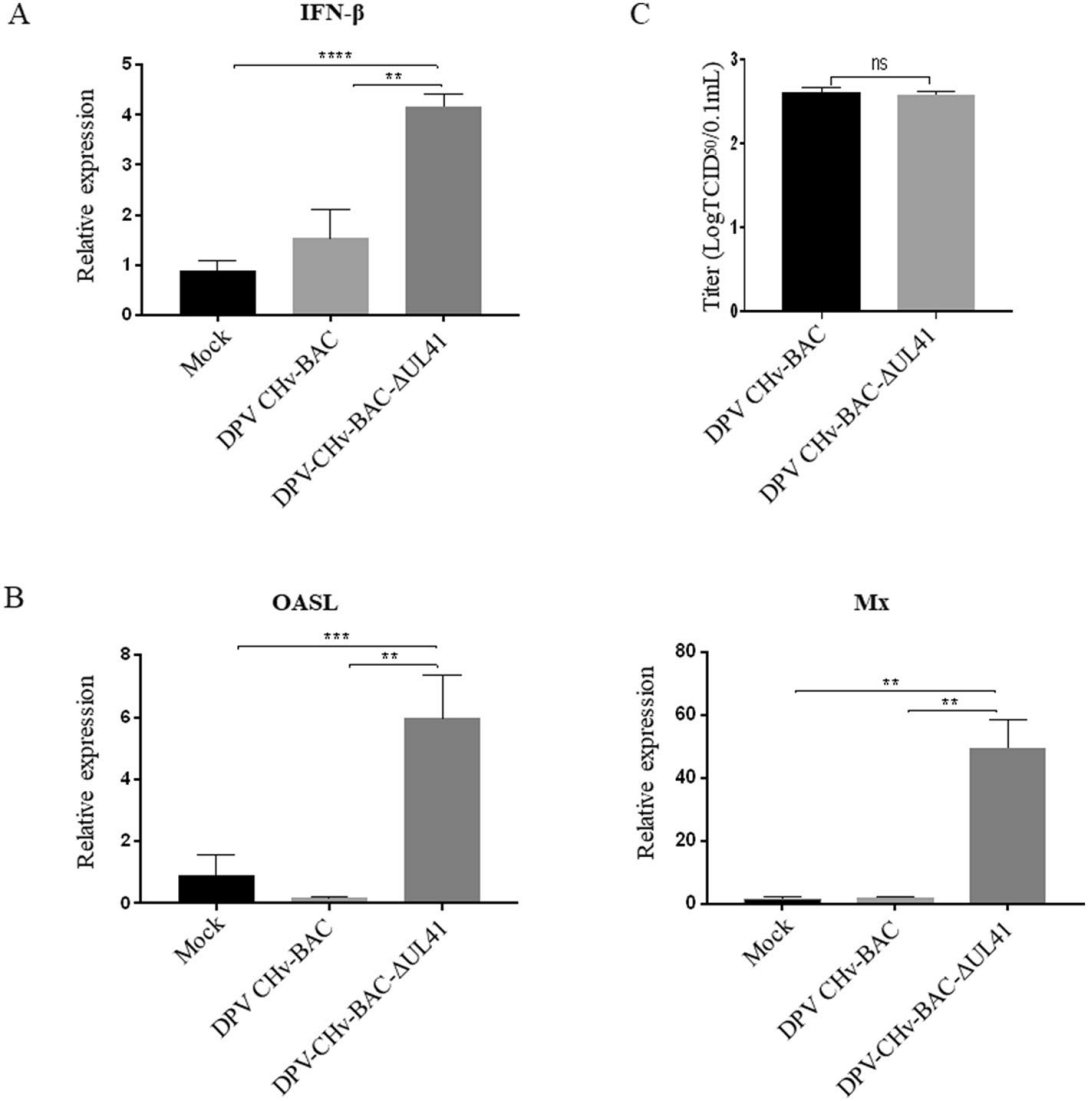
**DPV downregulated duck IFN-β and ISG production via the UL41 protein.** DEF cells were infected with the DPV CHv-BAC-G parent virus or the DPV CHv-BAC-G-ΔUL41 mutant virus at an MOI of 5 and then harvested and subjected to RT-PCR analysis at 4 hpi. **A** Quantification of IFN-β. **B** Quantification of Mx and OASL. **C** The viral titers in cytoplasmic samples were determined by TCID_50_. The data were analysed by one-way ANOVA. **p < 0.01, ***p < 0.001 and ****p < 0.0001.

These findings indicated that the DPV UL41 protein inhibits RIG-I/MDA5-mediated IFN-β production to promote viral infection by broadly affecting mRNA levels in vitro. DEF cells were infected with DPV CHv-BAC or DPV CHv-BAC-ΔUL41 and then collected at 4 hpi. We detected duck IFN-β and ISGs (Mx, OASL) production under viral infection. DPV CHv-BAC-G-ΔUL41 mutant virus infection induced more duck IFN-β and ISGs (Mx, OASL) production than infection with the DPV CHv-BAC parent virus. The viral titers in the cytoplasm did not differ between the DPV CHv-BAC-ΔUL41 mutant virus and the DPV CHv-BAC parent virus (Figure 6). These results further showed that the UL41 protein effectively inhibits duck IFN-β production.

### Knockdown of IRF7 expression increased the replication of the DPV CHv-BAC-ΔUL41 mutant virus

**Figure 7:**
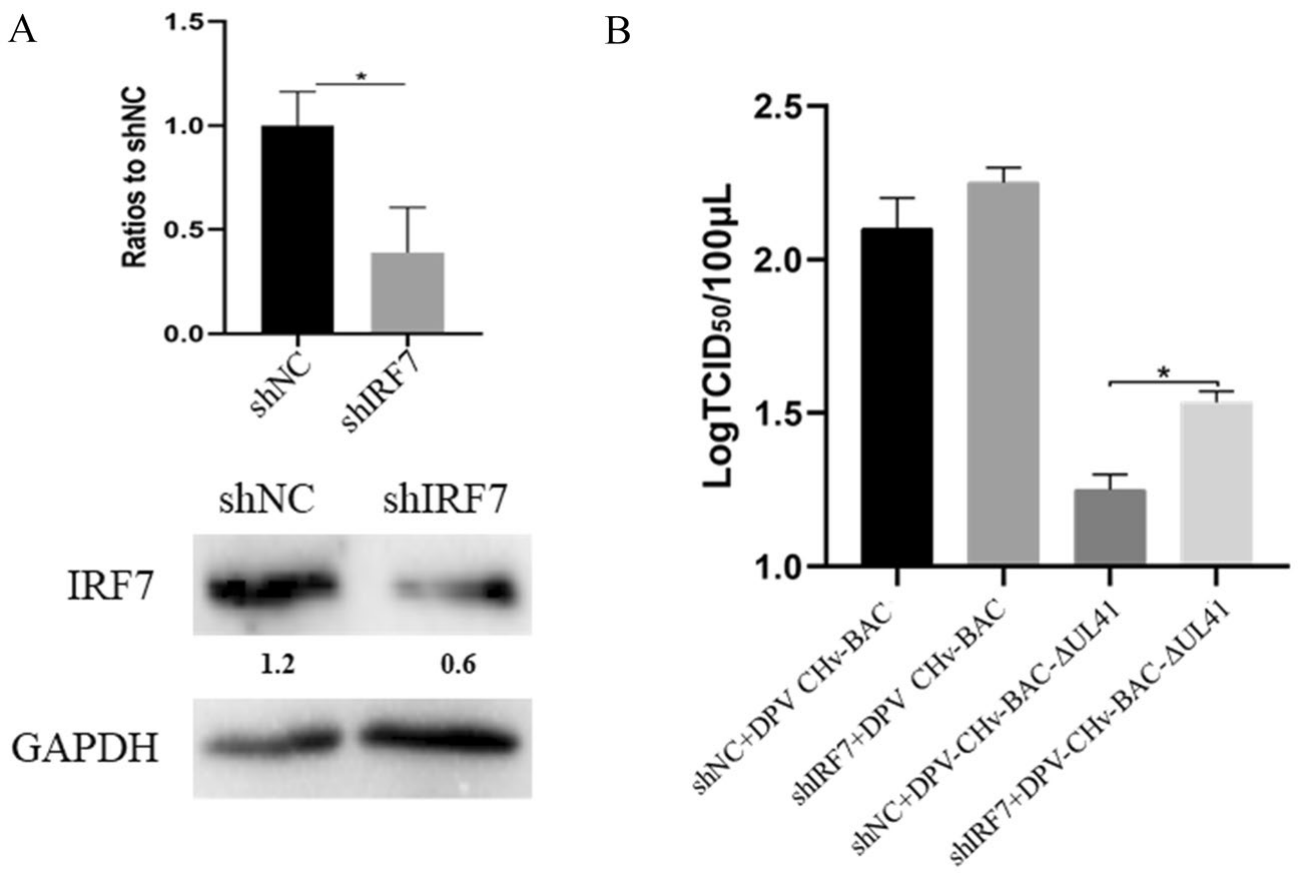
**Knockdown of IRF7 expression increased the replication of the DPV CHv-BAC-ΔUL41 mutant virus.** **A** DEF cells were transfected with pGPU6/GFP/Neo-shIRF7 or pGPU6/GFP/Neo-shNC, and the transcription and expression of endogenous IRF7 was detected by RT-qPCR and Western blotting, respectively, using a rabbit anti-IRF7 antibody (ABclonal, China, 1:1000). **B** DEF cells were transfected with pGPU6/GFP/Neo-shIRF7 or pGPU6/GFP/Neo-shNC and then infected with the DPV CHv-BAC parent virus or DPV CHv-BAC-ΔUL41 mutant virus at an MOI of 0.1 after 12 hpt. The viral titers were determined by the TCID_50_ at 24 hpi_._ The data were analysed by one-way ANOVA. *p < 0.05.

Because IRF3 is absent in the avian genome, IRF7 is an important adaptor and performs some of the IRF3 functions [50]. Previous studies reported that duck IRF7 activates IFN-I transcription and inhibits the in vitro replication of viruses, such as duck Tembusu virus (DTMUV) [51]. We knocked down the expression of IRF7 in DEF cells and examined the knockdown efficiency by RT-qPCR and Western blot. Compared with that in the shNC group, the expression of IRF7 was markedly downregulated in the shIRF7 group (Figure 7A). Then, we further detected the viral replication of the DPV CHv-BAC and DPV CHv-BAC-ΔUL41 recombinant viruses with IRF7 knockdown_._ As shown in Figure 7B, knockdown of IRF7 expression did not affect the replication of the DPV CHv-BAC parent virus but facilitated the replication of the DPV CHv-BAC-ΔUL41 mutant virus.

## Discussion

In this study, we reached three major conclusions. First, the DPV UL41 protein significantly inhibited duck IFN-β production. Second, the DPV UL41 protein inhibited the RIG-I/MDA5 innate immune pathway by broadly decreasing mRNA levels. Third, the conserved residues E229, D231 and D232 of the DPV UL41 protein were responsible for this activity.

Pattern recognition receptors (PRRs) sense viral nucleic acids or other virus-specific components, activating a series of signalling cascades to induce IFN and proinflammatory defence mechanisms in response to pathogens [52, 53]. RLRs function as cytosolic PRRs that initiate innate antiviral immunity by detecting exogenous viral RNAs. Significant amounts of dsRNA can be detected for viruses with positive-strand RNA, dsRNA and DNA genomes [54]. Therefore, RLRs also play an important role in the antiviral response to DNA viruses, such as Kaposi’s sarcoma-associated herpesvirus (KSHV) [55], Epstein-Barr virus (EBV) [56], murine gammaherpesvirus 68 (MHV68) [57], HSV [58] and DPV [36]. DPV infection activates high levels of RIG-I and MDA5 expression both in vitro and in vivo. Overexpression of RIG-I inhibits DPV infection, while its knockdown promotes DPV infection [36]. In addition, DPV infection was significantly suppressed in MDA5-overexpressing DEF cells, while the siRNA-mediated knockdown of MDA5 markedly enhanced DPV growth. LGP2 is a concentration-dependent switch that plays a role in MDA5-mediated antiviral activity against DPV [37]. These results collectively suggest that RIG-I and MDA5 act as anti-DPV molecules, and further studies are required to explore the molecular mechanism underlying the antiviral activity of RIG-I and MDA5 in ducks. However, all herpesviruses establish latent infections, a state from which the virus can be reactivated, resulting in recurring disease [59], and manipulation of the host immune response is required to accomplish this feat. KSHV four viral interferon regulatory factors (vIRF4) specifically inhibit IRF7 dimerization [60]. HSV-1 ICP27 targets the TBK1-activated STING signalosome to prevent IFN-I production [61], and the US11 protein binds to RIG-I and MDA5 to inhibit their downstream signalling pathway [62]. In this study, we first found that the UL41 protein inhibited duck RIG-I/MDA5-mediated duck IFN-β production via mRNA degradation activity. Infection with the DPV CHv-BAC-ΔUL41 mutant virus induced more duck IFN-β and ISGs (Mx and OASL) production than DPV CHv-BAC parent virus infection in DEF cells. Collectively, these results showed that DPV infection might evade immune responses via the UL41 protein.

VHS protein has been identified as an IFN-α/β resistance factor. HSV-2 VHS-deficient mutants induce > 50-fold more IFN-α/β than wild-type and VHS-rescued viruses in primary murine embryonic fibroblast (MEF) cells. HSV-2 VHS-deficient mutants are greatly attenuated in vivo, and replication and virulence are largely restored to the levels of the wild-type virus in mice lacking the IFN-α/β receptor [21, 63–65]. In addition, the HSV-1 VHS protein acts as a critical determinant of viral pathogenesis, and VHS-deficient mutants induce IFN and ISG production and increase susceptibility to IFN in cells [64]. However, the MDV *UL41* gene deletion mutant replicated in cell culture, and the degrees of tumour lesions and neurovirulence were equivalent to those of the lesions induced by the parental virus [66]. In this study, we found that the DPV UL41 protein induced increased, physiologically active levels of duck IFN-β and increased amounts of ISGs in DEF cells. Moreover, the DPV UL41 protein was shown to function as a duck IFN-β resistance factor to facilitate viral replication.

The VHS protein is an mRNA-specific RNase that evades the host innate immune response. The HSV-1 VHS protein directly degrades cGAS mRNA to downregulate IFN-β activation [67]. The HSV-2 VHS protein inhibits TLR2/3- and RIG-I/MDA5-mediated antiviral pathways [68]. The bovine herpesvirus 1 (BoHV-1) VHS protein does not affect TBK1- or IRF3-induced IFN-β production but suppresses the antiviral innate immune response by directly targeting the STAT1 transcript [69]. In this study, we first found that the DPV UL41 protein broadly abrogated RIG-I-, MDA5-, MAVS-, STING-, TBK1- and IRF7-mediated IFN-β-Luc activation and significantly inhibited the mRNA levels of these important adaptor molecules. Therefore, consistent with the UL41 proteins of other alphaherpesviruses, the DPV UL41 protein evades the host innate immune response by broadly regulating mRNA levels.

On the one hand, the VHS protein cleaves RNA on the 3’ sides of U and C residues in vitro [22]. The VHS protein degrades ribosome-associated mRNA by interacting with the cap-binding initiation factor in vivo [23]. Compared with the HSV-1 VHS protein, the DPV UL41 protein might cleave different mRNA sites to broadly degrade the molecules, but more experiments are required to explore this hypothesis. On the other hand, the VHS protein specifically degrades ARE-containing RNAs [70]. The HSV-1 VHS protein degrades cGAS mRNA, which contains three ARE core motifs (ATTTA) in the 3’ untranslated region (UTR) [67]. The BoHV-1 VHS protein binds the second ARE motif of STAT1 mRNA [69]. We also predicted the ARE motifs in the duck RIG-I, MDA5, MAVS, STING, TBK1 and IRF7 proteins and found they existed in the 3′UTRs of MAVS, STING, and TBK1 but not in that of RIG-I or IRF7. Therefore, we speculated that the mechanism by which the DPV UL41 protein degrades mRNA differs from that of the HSV-1 VHS protein. We also speculated that the DPV UL41 protein inhibits duck IFN-β production through other mechanisms. In addition, the HSV-1 VHS protein limits the accumulation of dsRNA [71]. We also speculated that the DPV UL41 protein directly destabilize dsRNA to downregulate the activation of important adaptor proteins in the RIG-I/MDA5 immune pathway.

The VHS protein and its homologues are present in only the *Alphaherpesvirinae* subfamily, and the VHS polypeptides of alphaherpesviruses are highly conserved [48]. A smaller but significant number of conserved residues, E192, D194, and D195, in the VHS protein were identified as crucial RNase active sites [50, 72]. We also identified and mutated the three conserved residues of E229, D231, and D232 in the DPV UL41 protein to alanine, and the mUL41 protein rescued duck IFN-β production and the mRNA levels of the important adaptor proteins. These results further showed that the active sites were highly conserved. In summary, this study is the first time to confirm that the DPV UL41 protein suppresses RIG-I/MDA5-mediated duck IFN-β production.

## Data Availability

The datasets analysed in this study are available from the corresponding author upon reasonable request.

## Abbreviations

DPV: duck plague virus
RIG-I: retinoic acid-inducible gene I
IFN-β: interferon-β
MDA5: melanoma differentiation-associated gene 5
MAVS: mitochondrial antiviral signalling protein
STING: stimulator of interferon gene
TBK1: TANK-binding kinase 1
IRF7: interferon regulatory factors 7
dsRNA: double-stranded RNA
VHS: virion host shutoff
HSV-1: herpes simplex virus-1
MHC: major histocompatibility complex
ISGs: IFN-stimulated genes
STAT1: signal transducer and activator of transcription 1
DEFs: duck embryo fibroblasts
FBS: foetal bovine serum
IFA: indirect immunofluorescence assay
KSHV: Kaposi’s sarcoma-associated herpesvirus
EBV: Epstein–Barr virus
MHV68: murine gammaherpesvirus 68
